# Successful Amputation in a Case of Gangrene

**Published:** 1854-12

**Authors:** 


### ART. II.—*Successful Amputation in a Case of Gangrene.*

By Drs. Jameson & Avery.

The result of the following successful amputation in a case of Gangrene, is perhaps sufficiently striking to merit publication, as tending to encourage operations in cases offering but little hope of success.

The patient, Joseph Baldwin, of Pitcher, who is about forty years of age, and usually in the enjoyment of good health, had, on the 21st of October, 1853, submitted to the removal of a large fatty tumor, occupying the calf of the leg and popliteal space. Nothing of note except the administration of chloroform and the securing of a dozen or more blood vessels, pertained to this operation. Nine days thereafter I saw him for the first time, in company with Dr. Jameson, who has preserved the following notes of the case:

"Oct. 30th. We were requested to visit Mr. Baldwin, with a view to the performance of amputation, the limb being reported as in a state of mortification.

On arriving at the patient's house, we learned from the attending physician that the foot had never regained its warmth since the operation, notwithstanding the use of the means commonly employed for that purpose; that in three or four days the foot exhibited decided marks of mortification, in which the leg, also, became rapidly involved. The whole of the leg to the knee, is now in a state of complete mortification: being cold, black, insensible, and exhaling a highly offensive and gangrenous odor. The thigh, in addition, is considerably tumefied, and the integument along the inner and posterior aspect as high as the upper third, presents a dusky redness, accompanied with hardness, some tenderness, and a blubbering sensation on pressure, which, when performed with the finger from above downward, occasion a copious discharge from the wound in the popliteal space, of a fœtid, sanguineo purulent fluid, rendering it manifest that the thigh as high as its upper third, has become seriously involved in the disease. The constitutional irritation is great; the tongue is coated; the pulse about 120; and he is exercised by a high degree of mental excitement; many think him delirious; but he still retains considerable strength, and being very desirous to have the limb removed, after the unfavorable aspect of the case had been fully presented to him; amputation in the circular form, was performed by Dr. Avery, as near the body as the application of the tourniquet would admit of, and the stump dressed in the usual manner. The administration of chloroform was not considered advisable in the exhausted condition of the patient. He was readily sustained, however, by the moderate use of brandy, and when removed to bed was in a very comfortable state. On examination of the diseased member after removal, suppuration was found beneath the integument of the thigh, and extending to within an inch of the incision. The patient was ordered an opiate at bed time, and a teaspoonful of brandy occasionally.

"31st. The patient has passed a tolerably comfortable night, having slept a little at intervals; took the opiate and two teaspoonfuls of brandy in course of the night; pulse 100, of moderate strength; has not had much pain in the stump, but considerable in the lumbar region; countenance rather indicates prostration; some bloody oozing from the stump, which exhales rather a gangrenous odor.

"Continue the brandy at short intervals, and let the patient have chicken broth and beef tea, 'ad libitum.'

"Nov. 1st. Seems as well as yesterday.

"Quinine, gr. i., every third hour, in a teaspoonful of brandy. Continue beef tea and chicken broth liberally.

"2d. Not quite so well; tongue a little more coated and disposed to dry in the center; pulse 112, and rather jerking; skin not particularly hot; took nearly an ounce of ol. ricini this morning, which has not operated yet; removed part of the adhesive straps from the stump, which, so far as could be seen, did not present any appearance of gangrene, although the dressings looked quite black and the discharge possessed considerable fœtor.

"Quinine, gr. i., every hour, in a teaspoonful of brandy. Continue beef tea, etc.

"3d. Doing well; bowels moved favorably; tongue disposed to clean; dressed remainder of stump, which, on the whole, presents a favorable appearance, the middle third having a tendency to slough; has no pain of it.

"Continue brandy and quinine, with strong nourishment.

"4th. Progresses favorably; a pledget of cotton dipped in chloride of soda, was applied to sloughy portion of the wound.

"Continue quinine, brandy, etc.

"5th. Sloughy part of wound appears better; tongue clean, but red and dry in the center; pulse 100, and rather jerking; some griping pain in the bowels, which have not been moved since the third.

"Continue medicine and diet as before; ol ricini, ℥i.

"6th. About the same; bowels not moved as yet; tongue clean, but dry and red; wound a little improved.

"Nitric acid lotion in place of chloride of soda. Continue med., etc.

"7th. Feels better; bowels moved last evening; tongue clean and moist; sloughy portion of wound cleaning, and healthy granulations springing up, the nitric acid lotion appearing to act well.

"From this date he rapidly improved under the same mode of treatment; nothing unusual occurring to interrupt his convalescence, further than a small abscess near the inner angle of the stump, which appeared to be connected with undue pressure of the bandage. The health of the patient is excellent. Three or four points in this case seem worthy of remark:

"*First.* The extent of mortification to the knee, and of suppuration to near the incision; together with the exhausted and irritable condition of the patient, and the favorable termination of the case.

"*Second.* Chloroform was withheld, as previous misfortune had taught us the extreme danger of administering the article in debilitated cases.

"*Third.* Perseverance in the use of brandy, quinine, and nourishment, notwithstanding apparent contraïndications. We are of opinion the stump would not have healed, nor would the patient have survived had he not been perseveringly nourished and stimulated."

